# Migrant Women’s Access to Sexual and Reproductive Health Services in Malaysia: A Qualitative Study

**DOI:** 10.3390/ijerph17155376

**Published:** 2020-07-26

**Authors:** Tharani Loganathan, Zhie X. Chan, Allard W. de Smalen, Nicola S. Pocock

**Affiliations:** 1 Centre for Epidemiology and Evidence-based Practice, Department of Social and Preventive Medicine, University of Malaya, Kuala Lumpur 50603, Malaysia; 2 International Institute for Global Health (UNU-IIGH), United Nations University, Kuala Lumpur 56000, Malaysia; zhie.chan@unu.edu (Z.X.C.); allarddesmalen@gmail.com (A.W.d.S.); Nicola.Pocock@lshtm.ac.uk (N.S.P.); 3 Maastricht Graduate School of Governance, Maastricht University, 6211 AX Maastricht, The Netherlands; 4 Maastricht Economic and Social Research Institute on Innovation and Technology (UNU-MERIT), United Nations University, 6211 AX Maastricht, The Netherlands; 5 Gender Violence & Health Centre, London School of Hygiene and Tropical Medicine, London WC1E 7HT, UK

**Keywords:** migrant health, access to health, sexual and reproductive health, contraception

## Abstract

Providing sexual and reproductive health (SRH) services to migrant workers is key to fulfilling sustainable developmental goals. This study aims to explore key informants’ views on the provision of SRH services for migrant women in Malaysia, exploring the provision of SRH education, contraception, abortion, antenatal and delivery, as well as the management of gender-based violence. In-depth interviews of 44 stakeholders were conducted from July 2018 to July 2019. Data were thematically analysed. Migrant workers that fall pregnant are unable to work legally and are subject to deportation. Despite this, we found that insufficient SRH information and contraceptive access are provided, as these are seen to encourage promiscuity. Pregnancy, rather than sexually transmitted infection prevention, is a core concern among migrant women, the latter of which is not adequately addressed by private providers. Abortions are often seen as the only option for pregnant migrants. Unsafe abortions occur which are linked to financial constraints and cultural disapproval, despite surgical abortions being legal in Malaysia. Pregnant migrants often delay care-seeking, and this may explain poor obstetric outcomes. Although health facilities for gender-based violence are available, non-citizen women face additional barriers in terms of discrimination and scrutiny by authorities. Migrant women face extremely limited options for SRH services in Malaysia and these should be expanded.

## 1. Introduction

Migration of women is an important component of international migration, with women comprising nearly half (48%) of the 258 million international migrants worldwide in 2017 [1]. In Malaysia, 19% of the 2.0 million documented migrant workers in 2019 were women [2]. The country also houses an estimated 2 to 3 million undocumented migrants [3], which increases the number of female migrants significantly. Although women represent a substantial proportion of less skilled migrant workers in Malaysia, appropriate migration and gender-sensitive policies are still lacking. As a result, female migrants are more vulnerable and prone to human rights violations [4,5].

Sexual and reproductive health and rights (SRHRs) are fundamental human rights, which lies with the right of individuals and couples to freely decide the number, timing, and spacing of children and have adequate information to make those decisions, and the right to attain the highest standard of sexual and reproductive health [SRH] [6]. SRHR was conceptualised during the 1994 International Conference on Population and Development (ICPD) in Cairo, where reproductive health was defined as a “state of complete physical, mental and social well-being, not merely the absence of disease and infirmity, in all matters relating to the reproductive system, and its functions and processes” [6] and was subsequently built on evolving international agreements [7].

SRH addresses a wide range of health issues, including contraception, unintended pregnancies, unsafe abortions, gender-based violence (GBV), pregnancy and childbirth complications, human immunodeficiency virus (HIV) and other sexually transmitted infections (STIs), and infertility and reproductive cancers, and are seen as essential elements to achieve social, economic and environmental development goals [7].

Maternal and child health services are the cornerstone of Malaysia’s public health system and are available nationwide as part of the integrated primary care services provided at public health clinics, maternal and child health clinics, and community clinics under the Ministry of Health (MOH), Malaysia. The scope of maternal and child health services includes maternal and perinatal health services (pre-pregnancy, antenatal, intrapartum and postnatal care), child health services (childhood immunisation and health, development and growth assessment), and woman’s health services (family planning services, and cervical and breast cancer screening) [8]. SRH services are also provided by the National Population and Family Development Board, under the purview of the Ministry of Women, Family and Community Development, non-governmental organisations like the Federal Reproductive Health Associations Malaysia (FRHAM), and private practitioners [9].

Improving access to SRH is central to development, as reflected under target 3.7 of the Sustainable Development Goals (SDGs) which calls for “universal access to sexual and reproductive health-care services, including for family planning, information, and education” by 2030 [10]. Although providing SRH services to marginalised communities including migrant workers are key to fulfilling the SDGs [11], the 2017 Voluntary National Review of SDGs by the Malaysian government did not identify migrant workers as a vulnerable group to improve delivery of healthcare services [12].

Women emigrating for employment face intersecting vulnerabilities of gender, social class, and ethnicity [13] and often encounter physical, psychological, and sexual violence [14,15]. Despite the ratification of the Convention on the Elimination of Discrimination against Women (CEDAW) and commitments to the ICPD Programme of Action [16], Malaysia has not fully recognised the migrant workers’ SRHRs [17,18]. Female migrant workers in Malaysia still face SRHR-related difficulties, mainly through the prohibition of pregnancy during employment [19,20].

In its concluding observations on the combined third to fifth periodic reports of Malaysia, the Committee on the Elimination of Discrimination against Women were concerned about the barriers faced by non-citizen women, including female migrant workers, when accessing healthcare [21]. Financial constraints are a major healthcare access barrier, as healthcare charges for non-citizens are considerably higher when compared to citizens for services at public facilities [17,18,22]. In addition, healthcare personnel are required to report undocumented migrants seeking medical care to the Immigration department, deterring women from seeking needed care due to fear of arrest and detention [23,24].

Therefore, this study aims to explore key informants’ views on the provision of SRH services for low-skilled, documented, and undocumented migrant women in Malaysia, including SRH education, contraception, abortion, antenatal and delivery, and the management of GBV.

## 2. Materials and Methods

We used qualitative methods in an exploratory, iterative design to explore policy and the provision of SRH services for migrant workers in Malaysia.

### 2.1. Definition of Terms

This study focuses on low-skill, low-wage migrant workers who cross international borders for employment. Documented or “regular” migrants are authorised to enter, stay, and partake in employment in a country, and also have legal documents, such as valid passports and work permits. Undocumented or “irregular” migrants are those who enter the country, reside, or partake in employment without authorisation, including those who may have entered the country legally, but have violated either the terms of their visa or over-stayed beyond the authorised period [9,10,11].

Refugees, asylum-seekers, foreign wives, and expatriates are not included in this study.

### 2.2. Sampling and Recruitment

We conducted 37 in-depth interviews with 44 individuals from July 2018 to July 2019 in Malaysia (Table 1). Most interviews were conducted on a one-on-one basis, while several were conducted in small groups of 2 to 3 participants from the same organisation.

The health and welfare of migrant workers in Malaysia are contentious, with issues concerning migrant workers’ SRHR and their immigration status being particularly sensitive. As such, we did not specifically target female migrant workers for interviews. We interviewed multiple stakeholders, including members of civil society organisations (CSOs), international organisations, academia, industry, medical doctors, and migrant representatives to obtain a broader understanding of SRHR for this vulnerable population.

Migrant representatives interviewed represented the interests of migrant workers and were able to speak broadly on migrant workers’ experiences in Malaysia. We interviewed representatives of workers from major migrant-sending countries to Malaysia (Indonesia, the Philippines, Nepal, and Bangladesh). We also interviewed medical doctors from the public sector, private sector, and CSOs, who provided SRH services to migrant populations. In addition, we interviewed representatives of CSOs that primarily worked on migrant women’s rights and welfare.

Participants were recruited purposively from a previous migrant health stakeholder workshop in Kuala Lumpur [25], and subsequently from snowball sampling of interviewees and further stakeholder recruitment through LinkedIn, until researchers agreed that new interviews would not yield additional information, as thematic saturation was reached.

### 2.3. Data Collection and Analysis

In-depth interviews averaged from 1 to 1.5 h and were conducted at physical locations chosen by participants or via telephone. Interviews were conducted either in English or Bahasa Malaysia (Malay language) depending on the participants’ preference, by the multi-lingual research team. The majority of interviews were conducted in English, with only 5 out of 37 interviews conducted in Malay.

Semi-structured interview guides were developed to seek participants’ perspectives on SRH health services for migrant women in Malaysia, and these questions were tailored towards the participants’ professional and organisational backgrounds. The interview guides were constructed based on literature review and discussion among the research team. Concurrent data analysis informed data collection and further refinement of question guides. Interviews with stakeholders from different backgrounds allowed triangulation of findings. Audio recordings were transcribed verbatim.

We conducted thematic analysis as described by Braun and Clarke, where themes or patterns of meaning within data were identified and reported using six phases: becoming familiar with the data, generating initial codes, searching for themes, reviewing themes, defining themes, and producing the report [26].

Data analysis was conducted in an immersive, exploratory, and inductive manner. The first and second authors reviewed and analysed transcripts independently, with regular discussions between researchers to refine codes and identify new themes. Transcripts were coded into emerging themes using NViVo 12 Plus, (QSR International, Melbourne, Australia) and quotations were extracted into Microsoft^®^ Excel^®^ for Office 365, (Microsoft, Redmond, WA, USA). Interviews in Bahasa Malaysia were analysed in the same language, while extracted quotations were translated to English for publication.

### 2.4. Reflexivity

Interviews were conducted by a medical doctor and academic researchers, who could be perceived as trusted authority figures. To counter possible power imbalances, especially among migrant workers and their representatives, participants chose interview times and their locations.

### 2.5. Ethics

Participant information sheets were distributed, which detailed the benefits and potential risks of the study, as well as patient rights and study procedures, including audio recording, confidentiality, and data storage. Verbal and written informed consent were sought from all participants before interviews. All participants agreed to be audio recorded and quoted anonymously in publications. Audio recordings and electronic transcripts were stored in secure data servers, while printed transcripts and notes were stored in a locked cupboard. Study participation was voluntary, and we explained that participants could refuse to answer questions or terminate interviews at any point.

This study was approved by the Medical Ethics Committee, University Malaya Medical Center, Malaysia and the Medical Research and Ethics Committee, Ministry of Health, Malaysia (Approval numbers: UM.TNC2/UMREC-238 and NMRR-18-1309-42043).

## 3. Results

Study results are presented on the health policy context, followed by findings on SRH services, such as SRH education and contraception, abortion, antenatal care and delivery, and GBV. Table 2 summarises the major study results.

### 3.1. Health Policy and Employment Contract Clauses

#### 3.1.1. Mandatory Health Screening and the Prohibition of Pregnancy

To obtain and renew work permits in Malaysia, documented migrant workers must undergo mandatory pre-employment medical examinations within the first month of arrival, and subsequently, annual medical examinations. These medical examinations are conducted at private clinics approved by the Foreign Workers Medical Examination and Monitoring Agency (Fomema) and include screening for a list of communicable and non-communicable diseases like HIV/AIDS, syphilis, tuberculosis, leprosy, hepatitis B, malaria, diabetes mellitus, hypertension, and also pregnancy for women. Female migrant workers testing positive for pregnancy will fail their medical examinations, and consequently will be denied work permits and are subject to deportation.

Most participants agreed that prohibiting pregnancy during employment is an infringement of a woman’s reproductive rights and is discriminatory against women. This migrant representative described how pregnancy is equated with illness in mandatory health screening.

*“The women who are pregnant, they are considered [as having] an illness. Pregnancy is an illness. They failed [the FOMEMA medical examination] and they have to be sent back. It is like they discriminate [against] us as a woman. This is our reproductive right.”*

*MW-1*

This interviewee expressed discomfort with the government-mandated screening for pregnancy, as it does not fulfil the purpose of a pre-employment medical examination to ensure “fitness to work” and to protect the public from communicable disease.

*“To get your work permit, you have to pass the medical screening, but the medical screening is not only screening for contagious disease, but also for pregnancy. For me, personally, it becomes a problem when it infringes the reproductive right [of migrants]. Other screenings make sense, that is something that is needed to ensure public health for everyone. For the workers themselves to be ‘fit to work’ and for the health of society, they have to be free from contagious disease–that makes sense! But, reproductive health issue–that concerns reproductive rights. It infringes human rights.”*

*CSO-8*

#### 3.1.2. Employment Contracts Prohibit Relationships, Marriages and Pregnancy

Several participants shared that employment contracts expressly forbid sexual relationships, marriages, and pregnancy. This medical practitioner explained that while both men and women are expected to be celibate, women are especially vulnerable because of the possibility of pregnancy.

*“Most of the migrant workers, especially the women, when they sign up agreements [employment contracts] with their companies, they are not allowed to get pregnant or be sexually active [throughout employment]. A lot of women have come to me and say, ‘My boss shouldn’t know this!’ Because you are not allowed to have sex. It doesn’t make sense! You are staying in this country for two years or more, and you are not allowed to have sex? Men and women are the same. But for the men, you don’t see much consequences because they don’t get pregnant! They don’t have to worry about getting pregnant! Women have a more vulnerable position because they fear they will get pregnant.”*

*MD-12 CSO*

The immediate termination from employment is a direct consequence of pregnancy. This stakeholder informed that this practice, while legal, is inherently discriminatory against women.

*“Migrant workers who are pregnant, they lose their job almost immediately. So, these are some of the concerns that people are afraid of… In terms of why is there a discriminative practice? If the woman is pregnant, you automatically lose the job. That is questionable.”*

*IO-2*

#### 3.1.3. Prohibiting Pregnancy Forces Women to Become Undocumented

Unlike expatriates from a professional, managerial, or highly skilled technical backgrounds, less skilled migrant workers are not allowed to bring family members or to get married in Malaysia in policy. This participant explains that this denial of the right to family results in unregistered marriages among non-citizens.

*“Reproductive rights, it is actually a basic of human right. You cannot say that [when] you come here, only the expatriate can have the family, non-expatriate cannot. This is human nature, you know? They got married, but they are not allowed to get married here. That is why there is a lot of ‘nikah bawah tanah’ [underground/unregistered marriages], so they get their own ‘imam’ [priest]…”*

*IO-1*

Participants explained that migrant women who are pregnant and opt to keep their babies are driven to become undocumented migrants. This medical practitioner expresses surprise that many migrant women opt to deliver their babies in Malaysia despite the severe consequences.

*“They will automatically be illegal migrants, because the moment they are pregnant, they will lose their visa and if they lose their visa, they become illegal migrants. But somehow, many of them do deliver locally.”*

*MD-9 PRIVATE GP*

### 3.2. Sexual and Reproductive Health Education and Contraception

#### 3.2.1. Employers do not Provide Access to Family Planning

Although pregnancy is prohibited among low-skilled migrant women employed in Malaysia in policy, those interviewed informed that there is little support from employers in terms of providing SRH education or services, either in terms of preventing STIs or providing family planning services. This interviewee explained that the prevalent moral attitude in Malaysia—that providing family planning encourages sexual promiscuity—may explain employers’ attitudes.

*“The thing that upsets me is that there is very little recognition that women migrant workers who come here are young and usually sexually active. It’s a fact of life. We have actually tried, through our NGOs, to promote the information on contraception, and access to contraception for these people. But people [employers] are very cagey about this! It all has got to do with the idea that: ‘Oh, they are only here to work, you know. They are not supposed to have boyfriends or relationships.’ And therefore, ‘Why should we give them any information on contraception? It will only make them bad workers.’ But the reality is, many of them are sexually active. And then, of course, if they don’t have access to contraception, they get unwanted pregnancies. And of course, for them to terminate their contract halfway, it’s a real waste because they made arrangements to do a two to four year contract with the factories, intending to earn and send money home. But the moment they are found to be pregnant, you know, they have two choices; They either have an abortion, or they are sent back.”*

*MD-9 PRIVATE GP*

This participant implied that providing family planning services is an important investment for both employers and workers, as unwanted pregnancies may result in job loss.

Nevertheless, civil society organisations have approached employers and embassies to provide SRH awareness for migrant workers with mixed success. This participant illustrates the best practices of multinational companies that invest in their employee’s health by training migrant community leaders to ensure the continuous education of new recruits.

*“There were programmes done by our NGO with a few companies, where we train their community leaders. So, we will start talking about, ‘What is the menstrual cycle? How to prevent STDs? About contraception and everything’. So, these community leaders will keep training new people [newly recruited migrant workers]. So, they know where to get contraception and will come to the clinic to get this [SRH services]”*

*MD-12 CSO*

#### 3.2.2. Migrants Pay Out-of-Pocket for Sexual Reproductive Health Services

Family planning is freely available to local patients at public clinics, as part of a comprehensive package of maternal and child health services for citizens. This medical doctor explained that financial constraints may deter some female migrants from seeking contraception at public clinics, as non-citizens must pay for services.

*“To be honest, migrants have to pay for the contraception-for injectable hormonal therapy or any sort of contraception-they have to pay! As opposed to locals, where contraception is free. So, the problem still comes back to financial issue. So, if they are willing to pay and can afford, and if they understand the importance to not conceive within the next two years, then they will pay for it. But most of them-no [they won’t pay].”*

*MD-13 PUBLIC CLINIC*

Migrant workers pay out-of-pocket for contraception at private clinics, as SRH services are not covered by the government-mandated migrant health insurance (SPIKPA) or employer-provided healthcare.

#### 3.2.3. Private Practitioners Promote Expensive Contraceptives and Fail to Provide Information on SRH

Medical practitioners interviewed informed that although a wide range of contraceptives are available at private clinics, most migrants prefer injectable hormonal contraceptives, especially the commonly available Depo-Provera injections. This interviewee explained that private doctors do not sufficiently advise women on contraceptive options, such as on the use of long-acting contraceptives like intrauterine contraceptive devices (IUCDs) or implants, because these options are less lucrative than injectable hormonal contraceptives.

*“Not many GPs [general practitioners] even want to talk about it! But they keep telling them to use Depo-Provera because it is profitable! In a year, if you are coming [to the clinic for] 4 times. So, RM 60 × 4 = RM 240. [Whereas, the] IUCD is RM 200 for 4 years. So, you are not going to see her for the next few years. It [the IUCD] is more economical for the woman, but it is less profitable for the doctors!”*

*MD-12 CSO*

While the private practitioners interviewed acknowledged that there is a substantial market for contraceptives among migrant women due to perceived need, participants explained that the awareness and willingness-to-pay are low for the prevention of STIs, specifically the use of condoms.

*“I got quite a number of them coming for depo injections [Depo-Provera injections]. Contraception, in the form to prevent pregnancy-yes. But to prevent STDs [sexually transmitted diseases], they have to buy la… Condoms and all that, they have to just find ways to buy it… But I have had quite a number of them who come in for depo injection. So, they do know about it, and they do come.”*

*MD-2 PRIVATE GP*

This participant implied that migrants were not willing-to-pay for condoms, as this was not seen as essential. Likewise, there is very little information provided by medical practitioners regarding the use of condoms in the prevention of STI.

### 3.3. Abortion

#### 3.3.1. Migrant Women’s Abortion Decisions Linked with Financial Security and Employer Support

Migrant women may lose formal employment and face deportation, as a consequence of pregnancy. Migrant women who chose to continue with their pregnancy in Malaysia are likely to become undocumented. Since the economic and social costs of pregnancy are substantial, this participant explained that migrant women that opt to continue with their pregnancy are usually in stable, committed relationships with relative financial security.

*“Migrants pay for antenatal care at private clinics themselves. So usually, the ones who are willing to keep a child, they know it’s going to cost them. So, they should have some ‘back up’ money or husbands who are ok, and then they can afford. Maybe he is taking home RM 1800 to RM 2000 a month. So, from all his work, he can afford it. Then they go ahead. There are some who will come and say, ‘No I can’t, I can’t afford it’. Then some are like girlfriend/boyfriend, but he might be married, she might be married, you know… ‘accidents’, you know. This group will come and ask if they can get a medical abortion.”*

*MD-2 PRIVATE GP*

Migrant workers are generally in Malaysia for the short term, with employment contracts lasting 2 to 4 years. This participant explained that many of the relationships formed by migrant workers in Malaysia are impermanent. Without support from a partner, pregnancies are unwanted and result in abortion.

*“Basically, when they arrive [in Malaysia], they may have a husband back home. But, after few months, no more. We heard from other Filipinos, that mostly after they separate from [their husband], they find someone else here. And then when they get pregnant, they just abort it.”*

*MW-3*

Several participants shared that some domestic workers are highly valued by their employers, and that these employers are supportive of their employees’ pregnancy. Examples were given of employers sending workers back to their home countries for delivery, with the option to return to Malaysia for employment. Others gave examples of employers supporting their workers by bringing them to private clinics for antenatal follow-up. This participant shared that some employers support their domestic workers in having an abortion, as this would mean the domestic worker could keep her job.

*“In terms of unwanted pregnancies, they cannot be pregnant and stay in the work. But fortunately, many of the private employers want to keep their maids. Very often their maids are quite well-paid and they [the employers] are happy with them. And if they are pregnant, the employer [would] actually bring her along [to the clinic], and then you [as the doctor], would do the termination because she wants to continue working.”*

*MD-9 Private clinic*

It was unclear whether domestic workers in this position received pre-abortion counselling or advice from providers on their options.

#### 3.3.2. Health Providers have Negative Attitudes towards Abortion

Although abortion is legal in Malaysia, the prevalent negative perception of termination of pregnancies has led to the widespread belief that it is illegal, even among healthcare providers. Many healthcare providers view contraception and abortion as sensitive topics and opt not to be part of the network of private healthcare providers offering safe abortion services. This participant explained that the opposition towards abortion is related to cultural norms, as the abortion laws in Malaysia are fairly liberal.

*“All of them who are at the top level [government] say: ‘Oh, yeah, we have to recognise the law.’ The law in Malaysia is almost identical to the English law on abortion. So, what happens on the ground, seems to be not so much an official policy, but all ‘cultural opposition’ to make reproductive health and particularly contraception accessible to single women, and to make safe abortion accessible to women in general.”*

*MD-9 PRIVATE GP*

Abortions are rarely conducted at public healthcare facilities. While a selected number of private clinics provide safe abortions, these options are expensive and maybe unaffordable for low-wage migrant workers. Thus, migrant women may opt to perform illegal, self-induced abortions, which are likely to be unsafe. Medical practitioners interviewed informed that migrant women do present at the emergency departments of public hospitals with complications of unsafe abortions.

*“I have never seen any migrants coming to us for abortions [at public clinics]. They do it by themselves–self-induced. They have their own traditional ways of doing it, you know, by drinking vinegar and certain traditional medication, or they will try to induce trauma to the stomach! So, when they do present to us, it is already–not there [pregnancy terminated]. So, we had to refer them to the [public] hospital for a D&C [Dilation and Curettage]. Curettage is to clear off whatever is left behind.”*

*MD-13 PUBLIC CLINIC*

#### 3.3.3. Medical Abortion Unavailable Legally

Malaysia has not legalised the use of medical abortions (i.e., non-surgical abortions, where oral medications are given to terminate pregnancy). While “abortion pills” are available for purchase online without a doctor’s prescription, these pills are illegal and unregulated. Medical practitioners interviewed explained that medical abortions are a safe alternative and cheaper than surgical abortions. This medical doctor cautions patients against purchasing “abortion pills” from unknown sources and would make discreet referrals to a professional network of CSOs that facilitates safe abortions.

*“We have to advise them on medical or a surgical abortion. A surgical abortion will cost them almost RM 700 to RM 1000, which most of them don’t have. So, instead of them harming themselves [unsafe abortion], we will actually tell them that ‘The procedure is not available here [at this clinic]. Don’t trust anybody, don’t Google, don’t find [abortion services] anywhere! Here are the contact details, where you can get pills online. But there’s a possibility of not fully recovering. You will then need to see these certain doctors [who are] providing surgical [abortion]!’ So, usually that is how we refer them to XXX.”*

*MD-12 CSO*

### 3.4. Antenatal Care and Delivery

#### 3.4.1. Migrants OPT for Private Clinics and Traditional Midwives for Antenatal Care

Due to immigration regulations, pregnant migrant workers in Malaysia inevitably become undocumented. While healthcare providers at public healthcare facilities will not deny patients necessary medical care, they are obliged to report undocumented workers to the police and immigration authorities. This medical practitioner explains that because of these restrictions, migrant women tend to opt for private healthcare.

*“They [migrants] tend not go to the ‘Klinik Kesihatan’ [public clinics for antenatal care], because they have to pay quite a bit for it. Some of them are scared that if they go there, and they [health authorities will] inform immigration department and they will be deported. So, they don’t want to go to the government side. So, they don’t get any [antenatal] follow up, they don’t get anything. Sometimes you [would] ask them, ‘Do you have antenatal records [home-based antenatal book given to patients at public clinics]?’ No records, you know, that makes it very difficult. But there are apparently some [private] clinics or some smaller maternity centres, who have their own follow-up for foreigners. So, they have their own [antenatal] book and they can go in for deliveries.”*

*MD-2 PRIVATE GP*

As the lack of antenatal follow-up and records prove problematic for the management of pregnancy and delivery, some more established private maternity centres provide more detailed follow-up for non-citizens.

Mainly due to the cost of private healthcare, some migrants prefer to deliver babies at home with the help of untrained traditional birth attendants. As shared by this migrant representative, this practice is likely done out of desperation, not cultural preference, and is linked with poor obstetric outcomes.

*“Some, they prefer to go to the traditional midwives. In some cases, that’s why they pass away during delivery, because they don’t want to go to the hospital. Because of the lack of documents and also because the payments are very high. So, they prefer to use the ‘dukun beranak’ [traditional midwife]. I found one [lady like that] last year, passed away in XXX. We had sent her to the hospital, but it was too late already. The baby also passed away.”*

*MW-1*

#### 3.4.2. Delayed Booking, Incomplete Antenatal Follow-Up and Poor Obstetric Outcomes

Most stakeholders explained that due to healthcare costs, non-citizens tend to present late for booking and default follow-up at antenatal clinics. Doctors interviewed observed that late presentations could result in poor obstetric outcomes and avoidable complications. These complications would inevitably incur additional financial expense, as more advanced treatment may be necessary.

*“For migrants, when they present, it is already 30 weeks? 32 weeks? I even had one patient last week [who] presented at 36 weeks! So, that was the first time ever that I saw her. So, whatever that has happened, has happened! It is irreversible. For example, that is something we called: IUGR, which is ‘Intrauterine growth restriction’. So, when that already occurs, nothing can be done! So, the baby may be born–with low birth weight from premature delivery. Then they will have a lot of complications! Like sepsis and all! So, all of these actually contribute to more financial burden to the patients! Because they will require a NIC [neonatal intensive care] admission for a long time!”*

*MD-13 PUBLIC CLINIC*

#### 3.4.3. Hospital Delivery Linked to Deportation

The government policy to report undocumented migrants to the police has resulted in incidents of women being detained immediately after delivery. Participants explained that conditions at detention centres are unsuitable for post-partum women and newborn babies. This interviewee explains that linking healthcare with deportation is a human rights violation.

*“We had a case of a migrant worker [who] was admitted to the hospital due to deliver. Within less than 24 h, both mother and the baby were already at the XXX Detention Camp. We [the CSO] needed to get intervention from the Embassy. They shouldn’t detain the baby inside there because there are not such facilities, and besides, the mother was still very fragile, and shouldn’t be detained. The Immigration Department persisted with their decision but [with] expedited repatriation. The Indonesian embassy refused to bear the expenses [of repatriation], so we [the CSO] had to find money for them. Because, the Indonesian government also has certain [financial] constraints. This was actually a very challenging situation for us.”*

*IO-1*

### 3.5. Gender-Based Violence

#### 3.5.1. One Stop Crisis Centre Linked with Police

One Stop Crisis Centres (OSCC) were established in Malaysia since 1996 to assist survivors of gender-based violence (GBV), to obtain comprehensive care from multiple agencies in a common venue. The OSCC are located at the emergency departments of Ministry of Health hospitals in Malaysia and provide immediate treatment, while facilitating protection, counselling, medico-legal, and social support services for survivors of GBV, rape, sodomy and sexual assault, domestic violence, and child abuse.

Legally, citizens and non-citizens can use GBV-associated healthcare services, which are available free of charge regardless of citizenship status at OSCCs. However, reporting violence to the police is a pre-requisite to seeking care at the OSCC. This participant explains that the procedure for reporting to the police is not always consistent. Survivors of violence are supposed to go directly to the OSCC, and the police report should be done at the hospital. However, some survivors are asked by hospitals to go to the police station first before coming to hospital for treatment.

*“I have heard different information, at different times. Previously, I have heard [that] people should just go to the emergency [department] and then be referred to the OSCC. Then the police report will be lodged there. So, the police will go [there], to take the report. But I have also heard another story when they go to the emergency [department] and want to be directed to the OSCC, and they were asked to lodge [a police] report first, before they come [into OSCC].”*

*CSO-8*

This participant explained that fear of the police is a hindrance faced by many non-citizen women seeking care or justice. Law enforcement personnel were described as lacking sensitization in dealing with GBV, and migrant women face additional discrimination.

*“I would say on the whole, there is definitely a lack of sensitisation amongst the police. I think in general, when it comes to gender-based violence, there is a lot of ‘victim blaming’ and those kinds of attitudes that are pretty pervasive. For non-Malaysian women, there is another layer of discrimination and some xenophobia. So, I think the quality of services is even lower for them! And then sometimes, if it is a situation where the employer has not done what they need to do to renew the work permit or the visa, then they might be afraid to go to the police because they can get reported to immigration! So, that is often a reason for women not to access help.”*

*CSO-7*

Study participants informed that undocumented migrants were particularly afraid to come forward to report incidences of violence, due to their immigration status.

#### 3.5.2. Limited Shelters for Non-Citizens

While government and CSOs provide shelters, those interviewed informed that there is a shortage of shelters specifically designed for survivors of GBV. Study participants informed that government shelters provided by the Welfare Department are general shelters, which may also house the homeless or elderly populations, and may lack comprehensive case management of GBV.

Furthermore, not all government shelters accept non-citizen women. Participants informed that government shelters only accept non-citizens that have been issued protection orders by the courts.

*“There are two types of shelters, shelters run by the NGO and then shelters by [the] government, especially [the] Women Ministry [Ministry of Women, Family and Community Development] and the Jabatan Kebajikan [Welfare Department]. But government shelters that takes migrants are limited to migrant workers who have already been given a protection order; after the case has been determined by [the] police and court. Let’s say the charges [pending] can be categorised as human trafficking, then… the person will be given a protection order or an interim protection order during the investigation. Only then, will they be put in the government shelters.”*

*CSO-8*

Participants informed that migrants may also be reluctant to obtain refuge and protection at shelters, as they would have to make a report with the police. As government shelters are limited for migrant women, CSOs are an important source of assistance, also providing legal aid and counselling.

## 4. Discussion

In Malaysia, female migrant workers are subject to regulation of their reproductive rights with pre-employment and annual screenings for pregnancy, and face termination from employment if found pregnant.

Premature dismissals from employment are financially detrimental to both employers and migrant workers. Nevertheless, we found that information and access to family planning are seldom supplied to migrant workers by employers and not provided for by the government [20]. The state and employers essentially deny that migrant workers are sexually active adults, with the intent of avoiding being seen as promoting promiscuity by raising the topic of SRH. This outdated approach must change towards a pragmatic one, whereby migrant workers, including men, are provided with education and access to low-cost contraceptives. The low contraception prevalence in Malaysia (33.1% for all methods and 23.3% for modern methods) compared to the global estimate for 2019 (48.5% for all methods and 44.3% for modern methods) [27,28] may be explained by social, cultural, and structural barriers and lack of knowledge on contraception [29,30,31,32].

Our findings suggest that the choice of contraceptive methods among migrants may be influenced by the perceived risk of pregnancy and its consequences borne by women; hence, female-controlled methods like injectable steroids may be preferable, with less uptake of male-controlled barrier methods like male condoms. Poor uptake of condoms may also be explained by a worrying lack of awareness of STD and HIV prevention [33,34].

Although Malaysia has relatively liberal abortion laws, its interpretation is subject to cultural and religious resistance in the predominantly Muslim nation [35,36]. The Penal Code Act 574 (revised 1997) Section 312 permits safe abortion if performed by a registered medical practitioner and the medical practitioner determines that continuance of the pregnancy endangers the life of the pregnant woman or harms her physical or mental health [37,38,39].

In 2014, a 24-year-old Nepali migrant worker who opted for an abortion for fear of losing her job was arrested when police raided the clinic where she had her abortion. This Nepali worker was the first woman charged and convicted for having an abortion in Malaysia, although her conviction was subsequently acquitted [40,41,42]. This case illustrates the plight of migrant women under restrictive immigration laws and labour practices, as even after her innocence was proved and despite being no longer pregnant, the Nepali worker was dismissed by employers for being a “bad role model” [43,44].

Many medical practitioners, especially public sector providers, have conservative views and exercise personal judgement that restricts a woman’s access to safe abortion [35]. While abortion services are available at certain private clinics, we found that financial constraints were a likely barrier for less skilled migrant women. Furthermore, the lack of information on where to obtain safe abortions and the underlying social stigma [35,45,46,47] are plausible drivers for migrant women to seek unsafe abortions, including unregulated medical abortions.

Medical abortion is a non-invasive, effective method for early pregnancy termination (within 49 days of the last menstrual period), that gives control to the woman rather than the healthcare provider [48]. Despite the recent classification of misoprostol and mifepristone as essential drugs–“where permitted under national law and culturally acceptable” by the World Health Organization [49,50], the Ministry of Health, Malaysia has yet to approve their use for medical abortions [36,37].

Notably, no participants undertook pre- or post-abortion counselling, either for their decision-making and feelings around abortion or on contraception post-abortion. We have no evidence for the latter in developed countries on increasing contraceptive uptake and acceptability [51].

Prohibition of pregnancy may result in avoidance of needed care due to apprehensions of job loss and deportation, and this may lead to treatment delays or unsafe abortions. It is accepted as a given by employers and healthcare providers that migrant women will want to terminate pregnancies so they can retain employment. Yet, the legal basis to prohibit pregnancy is unclear, as pregnancy as a clause for dismissal from employment is not specifically included in Malaysia’s Employment Act or the Immigration Act [52,53]. Women are effectively coerced by policy and employment contracts into abortions, and this may curtail their reproductive rights.

Migrant workers face complex barriers in accessing healthcare in Malaysia, including financial constraints, the need to present legal documents like passports and work permits at public facilities, language barriers, discrimination, and physical inaccessibility [23,54]. Immigration policies in Malaysia essentially deny maternal and child health services for migrant workers at public facilities. Previous studies support our findings that migrant women are late in initiating antenatal care, while many never attend antenatal clinics and have home births with untrained birth attendants [19,55]. These factors may lead to delivery complications, as migrant women only seek care when critically ill, necessitating more advanced and expensive care [56]. As seen in other settings, migrant women are at higher risk of poor obstetric outcomes, including increased maternal and neonatal mortality, as compared to local women [57,58].

Malaysia successfully lowered maternal mortality through health system strengthening and meticulous auditing of all maternal deaths, including non-citizen deaths, through confidential enquiry into maternal deaths (CEMD) [59,60,61]. Unfortunately, while non-citizen maternal deaths are investigated in Malaysia, maternal mortality among migrants are not captured in national statistics reported internationally [56,60], raising questions if definitive measures to reduce risk in this group have been attempted.

States have the sovereign right to govern migration within national boundaries. However, the detention of new mothers and their babies for immigration offence may conflict with international laws and conventions. According to the Bangkok Rules or “the United Nations Rules for the Treatment of Women Prisoners”, non-custodial measures are preferred to the detention of vulnerable pregnant women and minor children [62]. Malaysia has ratified both the CEDAW and the Convention on the Rights of the Child (CRC) [16] and is under obligation to provide reasonable care and cater to the special needs of pregnant women, breastfeeding mothers, and mothers with children in custody [63].

In Malaysia, OSCC provides integrated services for victims of GBV at public hospitals [64,65]. While services are available to all women, in theory, barriers remain in practice for non-citizen women. Migrant women, especially those with precarious legal status, are reluctant to report violence or seek medical treatment, for fear of arrest and detention for an immigration offence. Lack of uniform implementation, seen here with confusion regarding the need for victims to report violence at police stations before seeking treatment, is an example of a shortfall in service. We would like to stress the importance of gender-sensitisation training among law enforcement agents, in terms of improving gender sensitivity and reducing discrimination against vulnerable non-citizen women [44,66].

This study has several limitations. Due to the sensitive nature of this study, we had difficulties obtaining interviews with migrant workers, employers, and policy stakeholders. Nevertheless, we were able to triangulate study findings by interviewing diverse key informants, including medical doctors, representatives of civil society organisations, trade unions, and academia. While the qualitative nature of this study precludes the generalisation of findings, we were able to illustrate the landscape of SRH services for migrant women in Malaysia by examining different stakeholder viewpoints and perspectives. We were also unable to fully explore the management of STIs and HIV/AIDS among migrant populations, an important component of SRH which would need dedicated future study.

This study has several strengths. Ours is one of few studies in Malaysia that explore the access to SRH services among vulnerable female migrant workers. We hope that this work will provide a vital understanding of some of the barriers faced by this vulnerable population and opportunities for intervention. We suggest that future quantitative research be conducted to fill the gap in SRRH data in Malaysia disaggregated by citizenship, especially on contraceptive usage, abortion, utilisation of SRH services, and maternal mortality.

## 5. Conclusions

This study shows that the SRHR of migrant workers remains severely curtailed in Malaysia. Political will is necessary to revise restrictive immigration laws and labour policies to enable low-skill migrant workers to fulfil their SRHR. We suggest that instead of the discriminatory prohibition of pregnancy during employment, that all migrant workers are provided with access to SRH education and low-cost contraception by employers. All pregnant women, including non-citizens, should also have equal access to antenatal and delivery care at public healthcare facilities, and healthcare access should be decriminalised. A more inclusive, rights-based approach to healthcare access would have population-wide benefits, and this would put Malaysia towards the path of meeting the SDG target of 3.7 for universal access to SRH services.

## Figures and Tables

**Table 1:** Characteristics of the study participants (*n* = 44).

Participant Background	Label	No.
Medical Doctor	MD	
Public		4
Private		6
Civil society organisation		3
Civil society organisation	CSO	10
Industry	IND	5
Migrant worker ^1^	MW	4
International organisation	IO	4
Trade union	TU	3
Academia	AC	3
Other policy stakeholders ^2^	POL	2
Total		44

^1^ Only 1 of the 4 migrant workers interviewed identified himself as a worker only. Others were also members of civil society organisations (2) or trade unions (1). ^2^ Government or government-linked organisation.

**Table 2:** Summary of major study findings.

**Health Policy and Employment Contract Clauses**
Mandatory health screening and the prohibition of pregnancy is discriminatory towards women Less skilled migrant workers are not allowed to bring family members or allowed to get married in Malaysia Prohibiting pregnancy forces women to become undocumented
**Sexual and Reproductive Health Education and Contraception**
Employers prohibit pregnancy but do not provide access to family planning Providing information on sexual and reproductive health or access to contraceptive services is seen to encourage promiscuity according to prevailing attitudes Financial constraints may deter female migrants from seeking contraception Private practitioners tend to promote more expensive contraceptives, like injectable hormonal contraceptives Private practitioners fail to inform on the prevention of sexually transmitted infections or encourage the use of condoms
**Abortion**
Pregnancy has high economic and social costs to migrant women Migrant women who opt to continue with pregnancy are likely to be in a stable relationship Although abortion is legal in Malaysia, prevailing cultural norms and financial barriers force migrants to opt for unsafe abortions Medical abortion is illegal, but ‘abortion pills’ are pragmatically recommended by some healthcare providers for purchase online
**Antenatal Care and Delivery**
Migrant women opt for private care for antenatal care as public clinics report undocumented workers to the immigration department Some opt for traditional midwives as a result of financial barriers Delayed booking and incomplete antenatal follow-up may result in poor obstetric outcomes Hospital delivery discouraged as linked to immigration at public facilities
**Gender-Based Violence**
One Stop Crisis Centres established as a common venue for victims of gender-based violence to access care, is linked with law enforcement Law enforcement personnel lack sensitization in gender-based violence Migrant women face added xenophobia and fear when in using One Stop Crisis Centres, especially if undocumented Lack of shelters available for non-citizens and shelters have limited specialisation in gender-based violence
